# RNAi screens identify CHD4 as an essential gene in breast cancer growth

**DOI:** 10.18632/oncotarget.12646

**Published:** 2016-10-13

**Authors:** Carolina D'Alesio, Simona Punzi, Angelo Cicalese, Lorenzo Fornasari, Laura Furia, Laura Riva, Alessandro Carugo, Giuseppe Curigliano, Carmen Criscitiello, Giancarlo Pruneri, Pier Giuseppe Pelicci, Mario Faretta, Daniela Bossi, Luisa Lanfrancone

**Affiliations:** ^1^ Department of Experimental Oncology, European Institute of Oncology, Milan 20141, Italy; ^2^ Center for Genomic Science of IIT@SEMM, Fondazione Istituto Italiano di Tecnologia, Milan 20139, Italy; ^3^ Department of Molecular and Cellular Oncology, UT MD Anderson Cancer Center, Houston, TX 77030, USA; ^4^ Division of Experimental Therapeutics, European Institute of Oncology, Milan 20141, Italy; ^5^ School of Medicine, University of Milan, Milan 20122, Italy; ^6^ Biobank for Translational Medicine Unit, Department of Pathology, European Institute of Oncology, Milan 20141, Italy; ^7^ Department of Oncology, University of Milan, Milan 20139, Italy

**Keywords:** RNAi screen, epigenetic targets, breast cancer, CHD4, *in vivo* murine and human models

## Abstract

Epigenetic regulation plays an essential role in tumor development and epigenetic modifiers are considered optimal potential druggable candidates. In order to identify new breast cancer vulnerabilities and improve therapeutic chances for patients, we performed *in vivo* and *in vitro* shRNA screens in a human breast cancer cell model (MCF10DCIS.com cell line) using epigenetic libraries. Among the genes identified in our screening, we deeply investigated the role of Chromodomain Helicase DNA binding Protein 4 (*CHD4*) in breast cancer tumorigenesis. *CHD4* silencing significantly reduced tumor growth *in vivo* and proliferation *in vitro* of MCF10DCIS.com cells. Similarly, *in vivo* breast cancer growth was decreased in a spontaneous mouse model of breast carcinoma (MMTV-NeuT system) and in metastatic patient-derived xenograft models. Conversely, no reduction in proliferative ability of non-transformed mammary epithelial cells (MCF10A) was detected. Moreover, we showed that *CHD4* depletion arrests proliferation by inducing a G0/G1 block of cell cycle associated with up-regulation of CDKN1A (p21). These results highlight the relevance of genetic screens in the identification of tumor frailties and the role of *CHD4* as a potential pharmacological target to inhibit breast cancer growth.

## INTRODUCTION

Loss of function shRNA screening has become an invaluable tool in cancer research since this approach allows the identification of new genes essential in cancer maintenance and growth [1–20]. A variety of screens have been performed in different tumor types, some of which has lead to the identification of previously uncharacterized oncogenes that can be now considered potential candidates for targeted therapies [16].

Breast cancer is a heterogeneous disease, which displays diverse biological characteristics, clinical behavior and response to treatment [21]. In the context of this malignancy, the discovery of new therapeutic targets is essential since it remains the leading cause of cancer death among females worldwide. Epigenetic factors, mediating reversible changes at chromatin level, can regulate tumorigenesis, plasticity and heterogeneity of breast cancer cells [22–24], so that effective benefits of epigenetic-targeted therapy are currently investigated to obtain new-generation drugs [25].

The Mi-2/nucleosome remodeling and deacetylase (NuRD) complex regulates the transcription of genes involved both in normal development and in tumorigenesis, by modifying the chromatin structure through the activity of histone deacetylases [26, 27]. It has been recently shown that several members of the NuRD complex (*RbAp46, HDAC1, 2* and *MTA1-3*) stimulate breast cancer formation and metastases dissemination by interacting with steroid receptors, growth factor receptors, and other cell-type specific transcriptional pathways [28–32]. Another member of the NuRD complex, the Chromodomain Helicase DNA binding Protein 4 (*CHD4*), has been shown to activate cell cycle transition and DNA-damage responses through distinct mechanisms [33–35]. However to date, the role of *CHD4* in breast cancer progression has been poorly investigated.

Here, we used a human breast cancer cell line (MCF10DCIS.com), known for its capability of recapitulating the various stages of the malignancy when transplanted in an immune-compromised host [36] to perform an RNAi screen *in vivo* and *in vitro*. MCF10DCIS.com cells are endowed with metastatic potential and their tumorigenic features have been extensively characterized *in vivo* and *in vitro* [14, 36]. We applied a conventional RNAi screen to identify epigenetic vulnerabilities in breast cancer. To this end, an shRNA, lentiviral-based library composed of chromatin modifiers has been used, as previously described [1], and *CHD4* identified as a crucial gene in breast cancer development. We demonstrated that *CHD4* silencing inhibits tumor growth *in vivo* and proliferation *in vitro* by strongly reducing cell cycle progression in xenografts of MCF10DCIS.com cells, in transgenic, HER2-activated, mouse model and in patient-derived xenografts (PDX) of breast cancer.

## RESULTS

### *In vivo* and *in vitro* shRNA screens in a human breast cancer cell line

**Figure 1:**
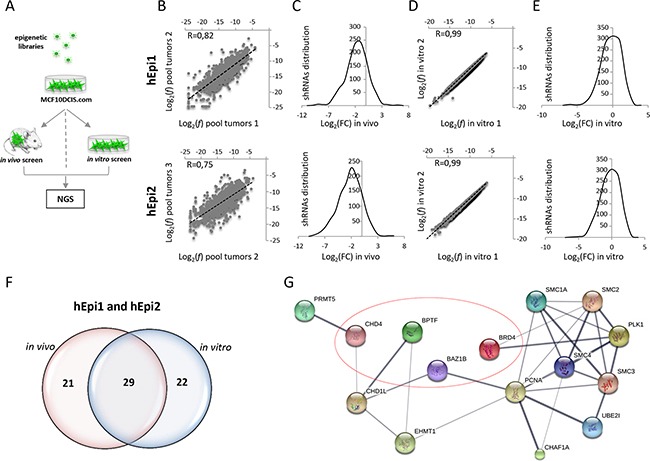
*In vivo* and *in vitro* shRNA screening using a human breast cancer cell line (MCF10DCIS.com) **A.** Graphical representation of the experimental procedure: MCF10DCIS.com cells were infected with hEpi1 and hEpi2 libraries and then orthotopically transplanted in the 4^th^ mammary gland of NOD/SCID mice (*in vivo* screen), or cultured *in vitro* for 21 days (*in vitro* screen). Genomic DNAs (gDNAs) were extracted from transduced cells as reference, tumors and *in vitro* cultures and subsequently subjected to PCR amplification and Next Generation Sequencing (NGS) to quantify shRNAs representation. **B.** Scatter plot representation of the shRNAs log_2_ frequencies (*f*) of two different pools (pool tumors 1 and 2) composed of four tumors each of hEpi1 (upper panel) and hEpi2 (lower panel) transduced cells. Black dotted lines represent the axis bisectors. Pearson correlation coefficient (R) indicates the similarity between the two samples. **C.** Distribution of the shRNA reads expressed as log_2_ Fold Change (FC) in the three pooled samples (mean of replicates) in hEpi1 (upper panel) and hEpi2 (lower panel) samples. **D.** Scatter plot representation of the shRNAs log_2_(*f*) of *in vitro* duplicates of hEpi1 (upper panel) and hEpi2 (lower panel) libraries. Black dotted lines represent the axis bisectors. Pearson correlation coefficient (R) indicates the similarity between the two samples. **E.** Distribution of the shRNA reads expressed as log_2_(FC) of two *in vitro* samples (mean of replicates) of hEpi1 (upper panel) and hEpi2 (lower panel) transduced cells. **F.** Venn diagrams reporting the number of genes scoring as depleted targets *in vivo*, *in vitro* and *in common* between the two settings. **G.** Protein interaction network of 15 out of 29 genes scoring by Ingenuity Pathway Analysis and significantly enriched for “Cell cycle” regulation analyzed in STRING. Line thickness represents the strength of data confidence. Encircled genes were selected for *in vivo* and *in vitro* validation of the screen.

To identify novel genes that sustain breast cancer growth, we performed loss of function *in vivo* and *in vitro* shRNA screens of epigenetic regulators in a human breast cancer cell line (MCF10DCIS.com). To investigate which epigenetic modifiers favor breast cancer growth, we used two custom pooled, barcode (BC)-coupled shRNA libraries composed of 1204 and 1192 shRNAs (hEpi1 and hEpi2, respectively), targeting 236 epigenetic regulators (118 in hEpi1 and 118 in hEpi2, see Materials and Methods for details) and four control genes (Luciferase - *LUC*, *KIF11*, *PSMA1* and *RPL30*, Supplementary Table S1) that were successfully used in an *in vivo* RNAi screen in melanoma [1]. MCF10DCIS.com cells were independently infected with the two libraries at low multiplicity of infection (MOI=0.2) so that each cell conceivably carried one single viral integrant. Ten different shRNAs were used to silence each gene. Transduced cells were either orthotopically injected into the mammary gland of immunodeficient mice (*in vivo* screen, 1.2^10^6^ cells/animal, four mice *per* replicate) or cultured *in vitro* (*in vitro* screen, 1.2^10^6^ cells/plate in duplicate), so that 1000 cells represented each single shRNA (Figure 1A).

Genomic DNAs extracted from transduced cells (reference), *in vitro* cultured cells and tumors grown *in vivo* were subjected to PCR amplification and Next Generation Sequencing (NGS) for barcodes (BCs) quantification, as previously described [1] (Figure 1A). In the library, each shRNA was univocally associated to a single BC.

We first analyzed the shRNA frequency and distribution in four tumors grown *in vivo*. Almost all shRNAs (around 98%) were recovered in the analyzed samples (data not shown) and the shRNA log_2_ frequency (*f*) showed high correlation between replicates (R> 0.71 for hEpi1 and R> 0.68 for hEpi2), suggesting that each tumor can represent the complexity of the whole library (Supplementary Figure S1A, S1B). We then compared the relative frequency of each shRNA *per* tumor to its respective reference and we calculated the log_2_ fold change (FC) of each library. The resulting distribution curves were shifted toward negative values (data not shown), suggesting that the epigenetic libraries exerted an inhibitory effect on *in vivo* breast cancer growth. To identify depleted genes (hits), we calculated the average of the z-score of the log_2_ FC of every single shRNA in the four tumors and assessed the distribution of the z-score values. We then considered depleted those shRNAs whose z-score value was equal or below the median of the curve and counted shRNAs depleted per gene (i.e. observed genes, Supplementary Figure S1C, S1D). To determine the minimum number of depleted shRNAs needed to score the hits and minimize the number of false positives, we applied a hypergeometric distribution, inferring the probability to find, on a set of 10 shRNA *per* gene (i.e. expected genes, Supplementary Figure S1C, S1D), the shRNAs scoring equal or below the median by chance (P=0.5) 0 to 10 times. We found that the “observed genes” overcome the “expected genes” when the depleted shRNAs are either 7 (hEpi1) or 8 (hEpi2) (Supplementary Figure S1C, S1D). To select candidate hits, we decided to set the cut-off threshold at 7 depleted shRNAs *per* gene (70% of the targeting shRNAs). We applied the same analysis to a pool of the above-mentioned tumors, and we observed that the resulting gene list corresponded well to that obtained by analyzing single tumors (data not shown), indicating that we can analyze tumors in pool. In order to obtain a biological triplicate, we sequenced and analyzed two more pools (4 tumors each). The scatter plots representing the shRNAs log_2_ (*f*) showed high correlation values among the *in vivo* pool replicates, ranging from R=0.82 (Figure 1B, upper panel for hEpi1) and R=0.75 (Figure 1B, lower panel for hEpi2), indicating a good experimental reproducibility even in a high heterogeneous system such as breast cancer. The log_2_ (FC) distributions of the shRNAs in the tumor pools (mean of biological triplicate) were shifted toward negative values, as expected (Figure 1C upper panel hEpi1 and lower panel hEpi2, respectively). Similarly, NGS analysis of the *in vitro* screen showed full representation of the libraries and a perfect correlation between the shRNA log_2_ (*f*) of the *in vitro* samples (R=0.99: Figure 1D, upper panel hEpi1 and lower panel hEpi2). The log_2_ (FC) distribution of the shRNAs in the *in vitro* duplicates (mean of replicates) followed a symmetrical distribution, as shown in Figure 1E (upper panel hEpi1 and lower panel hEpi2).

We generated a final list of candidate hits, considering depleted those genes whose single shRNA z-score values (70% of shRNAs *per* gene) were equal or below the median among the z-score distribution of triplicate pools *in vivo* or duplicates *in vitro*. The resulting list of depleted genes was composed of 50 and 51 genes (*in vivo* and *in vitro* respectively), 29 of which were in common between the two experimental conditions (Figure 1F). Notably, the positive control genes (*KIF11*, *PSMA1*, *RPL30*) and the neutral control (*LUC*) included in the epigenetic libraries scored as expected (Supplementary Table S1).

### Enrichment analysis of breast cancer hits reveals gene involvement in cell cycle regulation

We analyzed the list of the commonly depleted hits in the *in vivo* and *in vitro* settings by means of Ingenuity Pathway Analysis (IPA) and Molecular Signature Data Base (MSigDB) software to investigate if common pathways involved in tumorigenesis can be highlighted. IPA revealed that 27 genes (out of the 29 common genes) participated in the “cancer” signature (p-values: 4.96E-02 – 2.87E-05), which is consistent with the role of epigenetic targets in research and clinical practice. 15 out of 29 genes scored as significantly enriched in the “Cell cycle” regulation (*BAZ1B, BPTF, BRD4, CHAF1A, CHD1L, CHD4, EHMT1, PCNA, PLK1, PRMT5, SMC1A, SMC2, SMC3, SMC4, UBE2I*) (p-values: 4.13E-02 – 7.61E-09) (Supplementary Table S1) or “DNA replication, recombination and repair” (*BAHD1, BAZ1B, BPTF, BRD4, CHAF1A, CHD1L, CHD4, EHMT1, PCNA, PLK1, SIRT5, SMC1A, SMC2, SMC4, UBE2I*) (p-values: 4.05E-02 – 7.61E-09) signatures (Supplementary Table S1). Genes network representation of cell cycle regulators in STRING revealed high confidence connections between the majority of the hits (Figure 1G). MSigDB analysis confirmed the implication of our candidate hits in “Cell cycle related targets of E2F transcription factors” (p-value: 2.19E-9), “G2/M checkpoint” (p-value: 2.19E-9) and “Mitotic spindle assembly” (p-value: 6.57E-6) (Supplementary Table S1). Our newly identified candidate hits show a critical role in cell cycle regulation, and their modulation promotes the development of different types of cancer [33, 37–39], breast cancer in particular [40–44].

### *In vivo* and *in vitro* validation of the RNAi screens

**Figure 2:**
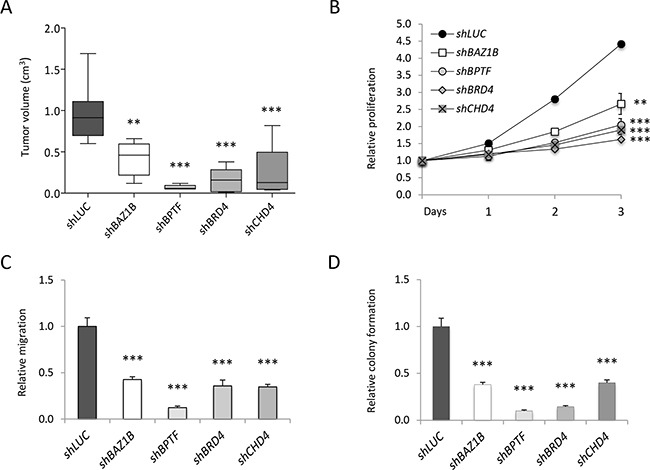
Validation of the shRNA screens MCF10DCIS.com infected with two pooled shRNAs targeting the indicated genes were used for *in vivo* **A.** and *in vitro* **B.** validation. A) Transduced cells were transplanted in NOD/SCID mice. Box plots represent tumor volume (mean±SD - cm^3^) of five to eight distinct tumors grown *in vivo*. B) Growth curves of shRNAs-infected cells were constructed calculating the relative proliferation values (mean±SD) expressed as ratio of the mean luminescent values in the shRNA expressing cells compared to the control (sh*LUC*) at time of plating (Day 0). Relative migration (mean±SD) **C.** and relative colony formation **D.** expressed as a ratio of silenced versus control (sh*LUC*) values, were calculated by ImageJ analysis. Statistical significances were calculated by applying the one-way ANOVA test followed by Dunnet's post-hoc test (**: P<0.01; ***: P<0.001).

In order to validate the *in vivo* and *in vitro* screens, we selected four candidates among the genes highly and concordantly depleted in the two screens (Supplementary Table S1). Bromodomain Adjacent to Zinc finger domain 1B (*BAZ1B*), Bromodomain PHD finger Transcription Factor (*BPTF*), Bromodomain containing 4 (*BRD4*) and *CHD4* are key components of various epigenetic complexes implicated in cancer growth, progression and/or metastasis formation [32, 37, 45–50]. MCF10DCIS.com cells were independently infected with two pooled shRNAs of the four candidates (shRNA#1 and #2) or control sh*LUC*. Silencing efficacy was measured using western blot analysis, as shown in Supplementary Figure S2A. Transduced cells were orthotopically transplanted in the mammary gland of NOD/SCID mice and tumor growth evaluated (*in vivo* validation). Depletion of each target significantly reduced (60-90%) the size of the tumors, as compared to control (Figure 2A), suggesting that all candidates have an oncogenic role in breast cancer. To validate our screening *in vitro*, shRNA-infected MCF10DCIS.com cells were analyzed for cell proliferation, migration and clonogenic abilities, all *in vitro* features associated with aggressiveness and metastatic potential [51]. Silencing of each gene caused a strong decrease of cell proliferation (40-70%) (Figure 2B), a robust reduction of migratory ability (60-80%) (Figure 2C), and a significant drop in the clonogenic capability (60-90%) of the cells (Figure 2D). Taken together, these results provide a robust validation of our screens, both *in vivo* and *in vitro*, suggesting that all selected hits are required for breast cancer growth.

### *CHD4* sustains tumor growth in murine and human breast cancer models

**Figure 3:**
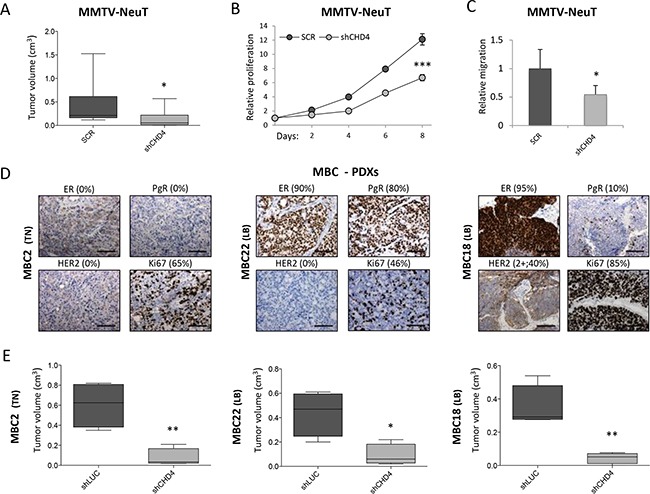
*CHD4* role in the MMTV-NeuT transgenic mouse and in PDX breast cancer models **A.** Cells derived from dissociation of spontaneous mammary tumors of MMTV-NeuT mice were infected with two pooled shRNAs silencing *Chd4* gene and a corresponding control (scramble – SCR) and subsequently transplanted in FVB mice. Box plots represent the tumor volume (mean±SD - cm^3^) of eight to ten distinct tumors grown *in vivo*. Statistical difference between groups was calculated using Mann-Whitney U test (U=14.0; *: P<0.05). Transduced cells were also used to analyze *in vitro* cell proliferation (eight days) **B.** and migration **C.** Statistical significance was calculated by applying a Student *t*-test (*: P<0.05; ***: P<0.001). **D.** Immunohistochemical staining of Estrogen (ER), Progesterone (PgR), HER2+ receptors and Ki67 in three metastatic breast cancer (MBC) patient-derived xenografts (PDXs). Percentage (%) of positive cells is reported for each staining. Scale bar: 100μm. **E.** Box plots representing tumor volume (mean±SD - cm^3^) of four distinct tumors arisen after transplantation of PDXs cells infected with a pool of two shRNAs targeting *CHD4* and the corresponding control (sh*LUC*) in NSG mice. Statistical significance was calculated by applying a Student *t*-test (*: P<0.05; **: P<0.01).

*CHD4* is a core component of the nucleosome remodeling and histone deacetylase NuRD complex, whose function can be exploited in combination with the other proteins of the NuRD complex, as well as alone [52]. To better uncover the role of *CHD4* in breast cancer maintenance, we chose the MMTV-NeuT transgenic mouse model that closely reflects some features of the aggressive human G3 breast cancer and of the human HER2 positive (+) tumors [53, 54]. Cells derived from the dissociation of spontaneously growing mammary tumors were infected using two pooled shRNAs targeting *Chd4* or control scramble (SCR) shRNAs and then transplanted into syngeneic mice. CHD4 protein levels were assessed at day 0 and 8 upon infection (at the beginning and the end of the proliferation assay) (Supplementary Figure S2B). *Chd4* silencing significantly reduced (about 67%) tumor growth *in vivo* (Figure 3A). As for the human cell line, we investigated the effect of *Chd4* inhibition on *in vitro* cell proliferation and migration (Figure 3B, 3C). Remarkably, knockdown of *Chd4* significantly reduced MMTV-NeuT cell growth (45%) and cell migration (46%) compared to the control (Figure 3B, 3C), confirming that *CHD4* is implicated in the development of HER2+ breast cancer, independently of the immunological context.

To investigate the effect of *CHD4* in a preclinical context, we developed a human metastatic breast cancer (MBC) model by direct implantation of patient-derived tumor tissue into the mammary fat pad of NSG mice to obtain a xenograft model (PDX). PDX cells were then serially transplanted and also grown in culture to obtain short-term human cultures. PDX tumors were stained for the most common prognostic markers, i.e. Estrogen (ER), Progesterone (PgR), HER2 receptors and Ki67, to assess the epithelial origin of the tumor, its correspondence with the patient tissue and its proliferative index (Figure 3D). PDXs phenotypically recapitulated the heterogeneity of the patient tumor (manuscript in preparation) and maintained their subtype classification (MBC2 is a Triple Negative and MBC22 and MBC18 are Luminal B breast cancers). Cells derived from dissociation of PDX tumors were infected with a pool of shRNAs targeting *CHD4* or neutral control (sh*LUC*), analyzed for silencing efficacy (Supplementary Figure S2C) and then re-transplanted in NSG mice. Notably, *CHD4* silencing significantly reduced *in vivo* growth (80%) (Figure 3E), confirming the role of *CHD4* in growth maintenance of two different metastatic breast cancer subtypes and suggesting that *CHD4* can be a potential druggable target also for the most aggressive diseases.

In order to evaluate the effects of *CHD4* inhibition on normal tissues, we studied *CHD4* silencing on the MCF10A human mammary epithelial cell line, representing the non-transformed counterpart of the MCF10DCIS.com cells, lacking tumorigenic potential *in vivo* and invasiveness *in vitro* [55]. MCF10A cells were efficiently silenced for *CHD4* (Supplementary Figure S3A) and transduced cells plated for *in vitro* assays, as done for the corresponding MCF10DCIS.com cancer cells. Silencing of *CHD4* did not significantly reduce proliferation (Supplementary Figure S3B), migration ability (Supplementary Figure S3C) and clonogenic potential (Supplementary Figure S3D), suggesting that depletion of *CHD4* should be likely effective in a cancer-specific context without influencing the biological and cellular functions of normal tissues.

### *CHD4* controls breast cancer cell cycle progression

**Figure 4:**
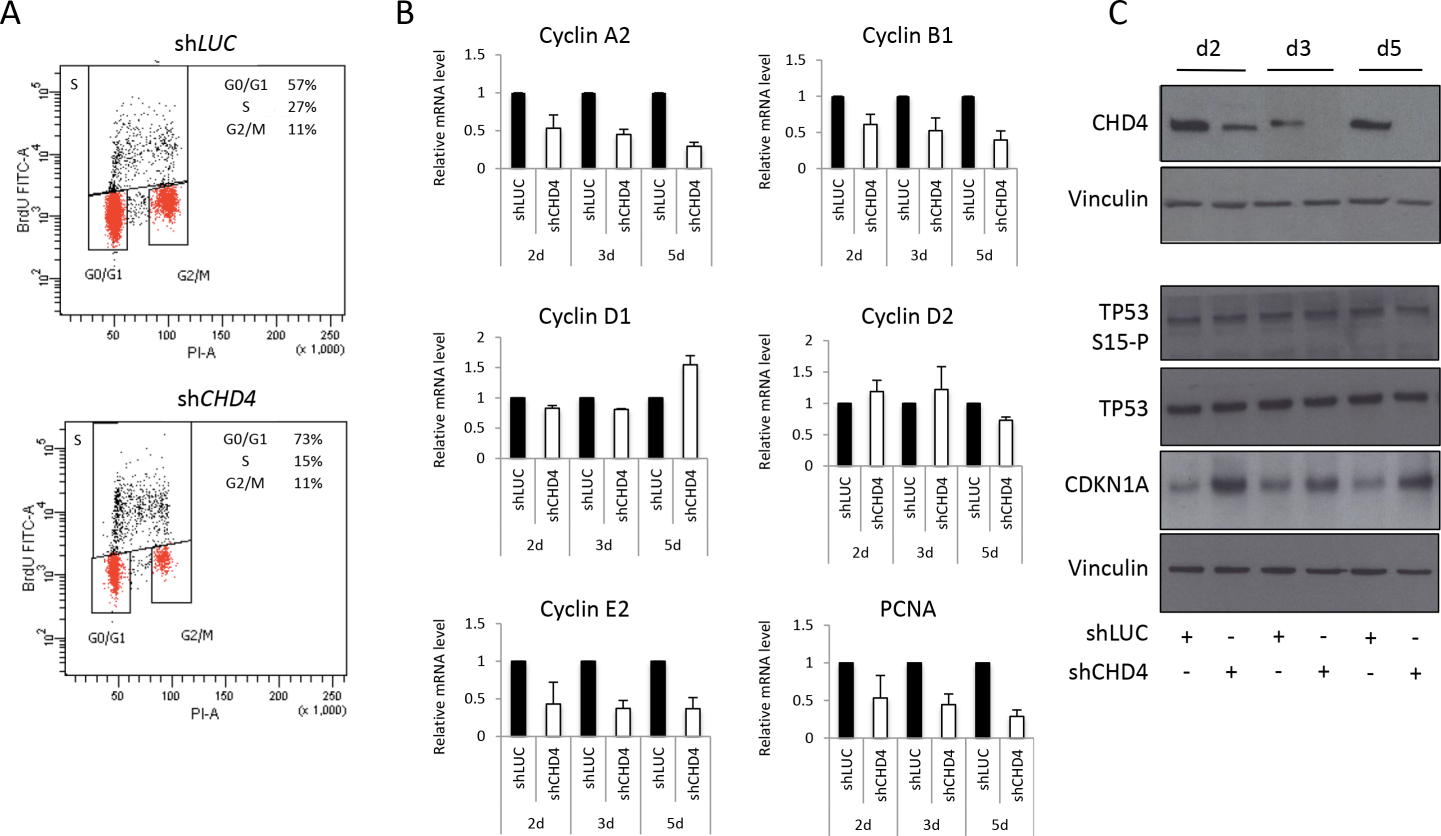
*CHD4* function in MCF10DCIS.com cell cycle progression **A.** MCF10DCIS.com cells were infected with sh*CHD4* or control (sh*LUC*) and after 5 days analyzed by flow cytometry for Bromodeoxyhuridine (BrDU) and PI content. Percentage (%) of cell population in each phase of cell cycle (G0/G1, S and G2/M) is reported within the panels. **B.** Effect of *CHD4* silencing on the regulation of different genes controlling cell cycle progression and proliferation was detected by qPCR analysis at different time points (days – d) from infection. Histograms represent mRNA levels (mean ± SE of two independent experiments) of cyclin A2, cyclin B1, cyclin D1 and D2, cyclin E2, PCNA in MCF10DCIS.com cell line. RPLP0 was used as housekeeper. **C.** Total TP53, phospho-TP53 (serine15) and CDKN1A levels were analyzed, at different time points (days – d) from infection, by western blot in MCF10DCIS.com cells infected with sh*CHD4* and the control sh*LUC*. Vinculin was used as normalizer.

*CHD4* knock-down can induce cell cycle arrest due to *CDKN1A* up-regulation in a *TP53* -dependent [33] or -independent manner [32], suggesting that it can differently regulate cell functions according to the biological context. To investigate *CHD4*-correlated mechanisms underlying the regulation of cell proliferation in breast cancer, we decided to examine the cell cycle progression of *CHD4-*silenced MCF10DCIS.com cells. Flow cytometric analysis revealed that *CHD4*-knock-down cells prevalently accumulate in the G0/G1 phase of the cell cycle (73%) compared to control cells (57%), with a consistent reduction of the cell population in the S phase (15% in sh*CHD4* cells *vs* 27% in sh*LUC*) (Figure 4A). Cell cycle deregulation is one of the hallmark of cancer, occurring through the alteration of proteins that influence cell cycle progression at different levels [56]. *CHD4* can stimulate a number of cell cycle regulators, including cyclins implicated in the G1/S transition or mitosis (cyclin A2, B1 and E2) and in the G1 phase activity (cyclin D1 and D2), and the checkpoint activation genes *TP53* and *CDKN1A* [57]. Firstly, we investigated cyclin levels at different time points (2, 3 and 5 days after infection) in MCF10DCIS.com transduced cells. QPCR analysis showed that *CHD4* depletion decreased mRNA levels of cyclin A2, B1 and E2, whereas cyclin D1 and D2 levels were not impaired (Figure 4B). PCNA mRNA was also reduced to a level similar to cyclin A2 and B1, consistently with the observed proliferation arrest (Figure 4B). It is known that *TP53-CDKN1A* axis directly and indirectly regulates cell cycle progression [58] and controls G1/S transition modulating cyclins levels [59]. Western blot analysis performed in MCF10DCIS.com transduced cells revealed that the reduction of *CHD4* did not increase TP53 protein level and its Serine-15 phosphorylation at different time points (2, 3 and 5 days after infection), but it determined a substantial up-regulation of CDKN1A protein level (Figure 4C) since the early time point (2 days since infection) of the experimental assay. Taken together, this data suggest that *CHD4* silencing increases CDKN1A content, decreases A2, B1 and E2 cyclin levels and arrests the cells in the G0/G1 phase of cycle progression rather than inducing apoptosis through the activation of caspases (data not shown).

**Figure 5:**
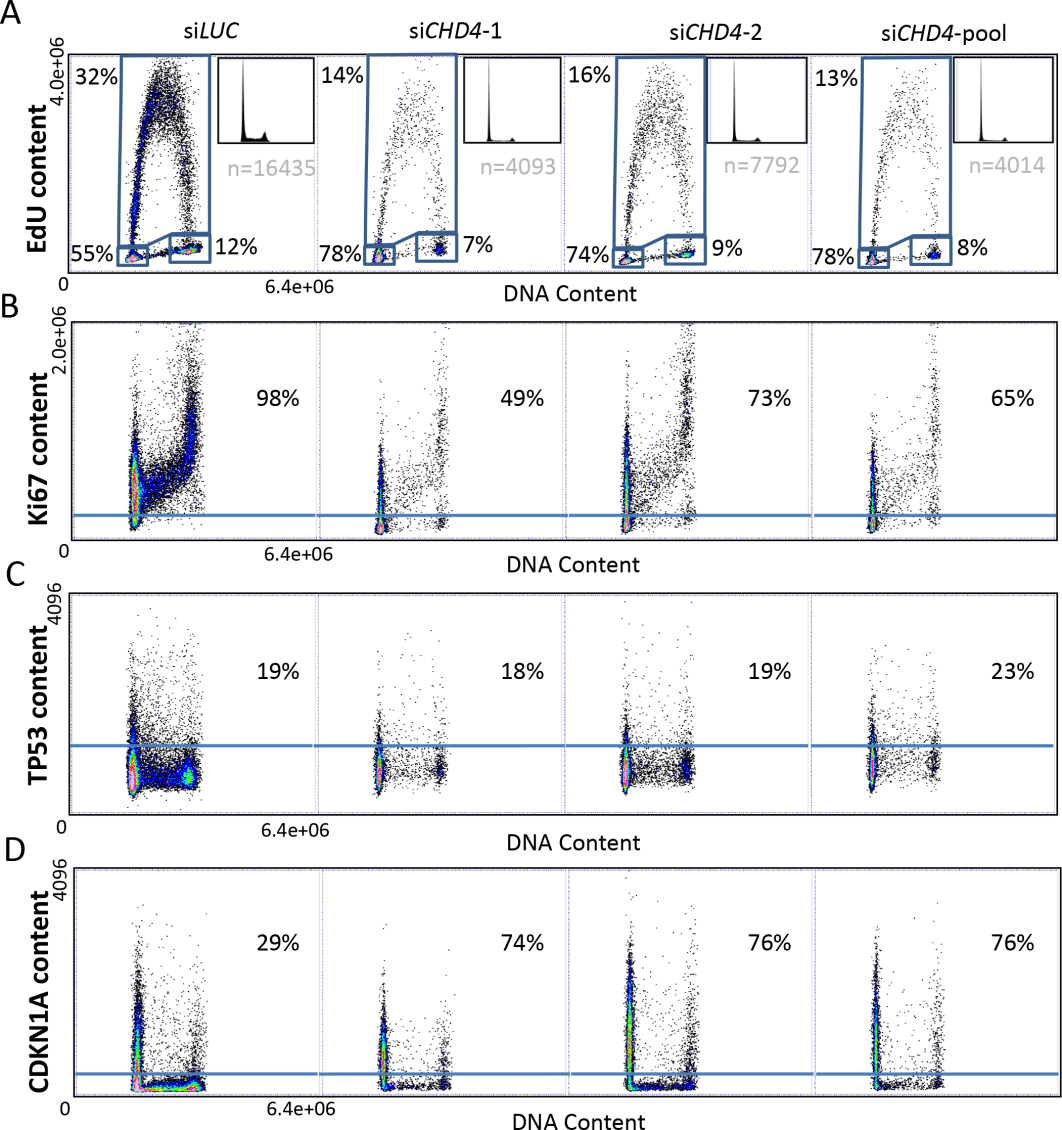
Cell cycle progression, proliferation index and checkpoint activation analysis by high-content and high-resolution multiparameter image cytometry MCF10DCIS.com cells were transfected with two separate (si*CHD4*-1 and si*CHD4*-2) or pooled (si*CHD4*-pool) siRNAs against *CHD4* or the control (si*LUC*). DNA content (x-axis) was correlated to the analyzed parameter (y-axis): EdU **A.** Ki67 **B.** TP53 **C.** CDKN1A **D.** content. Percentages of cells in each phase of cell cycle are reported with respect to the total cell population (A). Ki67 (B), TP53 (C) and CDKN1A (D) content are expressed as percentage with respect to the gated cell population.

To contextually analyze the effect of *CHD4* and its putative effectors in breast cancer cell cycle progression, we took advantage of a more quantitative and sensitive technique, the A.M.I.C.O. (automated microscopy for image cytometry) technology, which allowed us to perform a multi-parameter analysis, targeting specific cell subpopulations [60, 61]. To analyze effects of *CHD4* downregulation on proliferation ability, cell cycle progression and regulation we simultaneously assessed the level of i) *CHD4*, ii) Ethinyl-deoxyUridine (EdU), for active DNA synthesis detection, iii) Ki67, as a proliferation marker, and iv) *TP53* and *CDKN1A*, to monitor checkpoint activation, in relation to DNA content. We transiently transfected MCF10DCIS.com cells with two different siRNAs targeting *CHD4* (si*CHD4*-1 and si*CHD4*-2), the pool of the two siRNAs (si*CHD4*-pool) or siRNA Luciferase (si*LUC*) as control. We first confirmed that *CHD4* was efficiently silenced evaluating its content with respect to DNA in different cell populations (si*LUC*, si*CHD4*-1, si*CHD4*-2, si*CHD4*-pool). *CHD4* transfected cells showed low *CHD4* levels in comparison to neutral controls (Supplementary Figure S4A, S4B). We evaluated DNA profiles and DNA synthesis by EdU content analysis in MCF10DCIS.com cells confirming a robust cell cycle arrest in the G0/G1 phase of *CHD4*-silenced cells (Figure 5A). Furthermore, loss of *CHD4* significantly reduced the S phase of breast cancer cells (16-19%) (Figure 5A). The effect of *CHD4* knock-down on proliferative ability of the cells was further confirmed by the evaluation of the number of acquired events measured as cellular density in all conditions examined. In fact, a lower number of events were acquired for *CHD4* silenced cells due to a reduced proliferative capability (Figure 5A). The effect of *CHD4* knock-down on proliferation was further confirmed by the evaluation of the number of acquired events, as a measure of cellular spatial density. In fact the imaged area being equal, a lower number of events was acquired for *CHD4* silenced cells due to a reduced capability of these cells to proliferate (Figure 5A). In agreement with the above observation, the analysis of Ki67 content in the cells interfered for *CHD4* (Figure 5B) revealed a drastic reduction (25-49%) in the actively proliferating fraction compared to control population. Arrested cells were blocked in the G0 phase [62] with a complete exit from cell cycle well before the completion of the G1 phase.

Quite strikingly, analysis on *TP53* content revealed no modification of *TP53* level upon *CHD4* silencing in comparison to control cells (Figure 5C), confirming that *TP53* is not required to maintain G0 arrest. As a further confirmation of this data, the *CHD4* knocked down population was subdivided in *CHD4* positive (*CHD4*+, with *CHD4* residual expression) and negative (*CHD4*-) cells. The analysis of *TP53* content showed a similar distribution in both subpopulations (Supplementary Figure S4C).

Finally, analysis of *CDKN1A* levels in MCF10DCIS.com cells revealed a strong increase of *CDKN1A* content (45-47%) in the *CHD4* depleted cells, compared to neutral control (Figure 5D). Almost all silenced cells (*CHD4*-) showed a dramatic up-regulation of *CDKN1A* in comparison to *CHD4*+ cells (Supplementary Figure S4D) confirming that the down-regulation of *CHD4* causes a consistent cell cycle arrest and a consequent proliferation loss due to a significant up-regulation of *CDKN1A* level.

## DISCUSSION

Despite impressive improvements in breast cancer survival over the last decades, a not negligible number of patients still relapse as consequence of resistance to conventional treatment [63]. Therefore, the identification of new druggable genes involved in tumorigenesis must be considered of utmost importance. Here we show that we have identified novel epigenetic targets by means of an RNAi screen performed *in vivo* and *in vitro* in the MCF10DCIS.com cell line.

We took advantage of a published protocol in which MCF10DCIS.com cells were screened with a metabolic library composed of 516 shRNAs [14]. Although we increased the number of shRNAs composing each library (see Materials and Methods for details), we were able to represent the complexity of the system and contextually reduce the possibility of detecting false positive depleted genes, as more shRNAs were included in the analysis.

Our *in vitro* and *in vivo* approach allowed us to compare the two conditions and take into considerations those genes whose activity interferes with tumor growth either *in vivo* or *in vitro.* Approximately 50% of the genes that we found depleted in the two screens were common to the two conditions, suggesting that the regulation of epigenetic pathways in breast cancer relays on mechanisms that can be uncovered in the two contexts. Moreover, IPA showed that a relevant proportion of these genes are implicated in the control of cell cycle progression.

We have fully validated the epigenetic screens *in vivo* and *in vitro* by analyzing four different hits and we have demonstrated that *BAZ1B*, *BPTF*, *BRD4* and *CHD4* are essential for breast cancer growth. In particular, it has already been demonstrated that *CHD4*, together with the other subunits of the NuRD complex, is implicated in tumor progression [32, 48, 64], but up to now no data were available in breast cancer. In our system, *CHD4* silencing significantly reduces cell proliferation and migration both *in vivo* and *in vitro*, suggesting that *CHD4* inhibition can be important to block cancer progression.

Because of the complexity and heterogeneity of breast cancer, it seems crucial to set up appropriate preclinical systems to fully investigate the different aspects of this disease. Therefore, an integrated and multi-systems approach is currently the strongest way to model this disease and to study gene vulnerabilities [65].

Since the human *in vivo* shRNA screen has been performed in immunodeficient animals, we investigated the role of *CHD4* in a model in which tumor develops in the presence of an intact immune system. *In vivo* and *in vitro* silencing of *CHD4* in the MMTV-NeuT model revealed that it plays a crucial role also in a fully immune-competent system, meaning that the tumorigenic function of *CHD4* can bypass the intrinsic immune surveillance. Concerning the oncogenic role of *CHD4* in HER2+ breast cancer, it seems likely that targeting *CHD4* in HER2+ patients can be a valuable strategy to overcome resistance to approved drugs [66–68]. To explore the role of *CHD4* in patients, we used PDX models of Luminal B and Triple Negative breast cancer. The dramatic reduction of tumor growth induced by the loss of *CHD4* is extremely relevant and suggests that the pharmacological inhibition of this gene could improve the treatment of the most aggressive breast cancer subtype.

Despite the high selectivity of targeted therapy, unpredictable side effects and toxicity in normal cells can emerge [69]. Therefore it is extremely important to test the effects of *CHD4* silencing on normal cells. We showed that *CHD4* depletion did not influence the proliferative, migratory and clonogenic potential of the non-transformed MCF10A cells, suggesting that *CHD4* is selectively responsible of cell survival and proliferation in cancer cells only.

With this work, we shed light on the mechanisms through which *CHD4* stimulates breast cancer cell proliferation. Noticeably, for the first time, we show that *CHD4* is an essential gene in breast cancer progression. In particular, cell cycle analysis showed that the loss of *CHD4* causes MCF10DCIS.com cells to arrest in G0 phase, with a dramatic reduction of proliferation and a striking reduction of DNA synthesis. Loss of *CHD4* arrested the cells well before the G1/S transition as demonstrated by the selective loss of Ki67 proliferation marker. Differently to U2OS cells, where cycle progression is regulated in a *TP53*-dependent manner [33], our results suggest that *CHD4* can suppress cell cycle progression through *CDKN1A* up-regulation in breast cancer cells.

It has been shown that oncogenic *RAS*, as well as *RAF*, one of its downstream effectors, activates *CDKN1A* transcription through both *TP53*-dependent and *TP53*-independent mechanisms, the second one requiring the transcription factor *E2F1* [70]. MCF10DCIS.com cells contain an active *HRAS* and for this reason, the *E2F1* binding activity, as well as *HRAS*/*CHD4*/*E2F1* axis, will be actively investigated in these cells.

In conclusion, our approach identifies diverse epigenetic targets as crucial oncogenes in breast cancer, suggesting in particular that *CHD4* targeting can be used as an efficient strategy to arrest breast cancer progression.

## MATERIALS AND METHODS

### Libraries, plasmids and siRNAs

#### Libraries

Human epigenetic libraries were purchased from Cellecta Inc. and engineered into the pRSI-U6-(sh)-UbiC-GFP-2A-Puro lentiviral vector containing the puromycin-resistance and the GFP fluorescent marker. shRNAs were under the control of a constitutive U6 promoter and univocally associated to a barcode cassette (BC) of 18 degenerated, non-overlapping nucleotides. The libraries contained 1204 (hEpi1) and 1192 shRNAs (hEpi2) targeting 118 (hEpi1, 10 different shRNAs *per* gene) and 118 (hEpi2, 9 or 10 different shRNAs *per* gene) epigenetic genes, three positive (*KIF11, PSMA1, RPL30*) and one neutral (Luciferase, *LUC*) controls.

#### Plasmids

Each shRNA was cloned into the pRSI-U6-(sh)-UbiC-TagRFP-2A-Puro vector (Cellecta Inc.) and as pool of two shRNAs was used to infect target cells. Complete sequences of shRNAs used for validation experiments are reported in Supplementary Table S1. shRNAs targeting mouse genes were engineered into the pLKO.1 vector (Sigma). 3 scrambles shRNAs were pooled together and used as neutral control (SCR). The shRNAs targeting *Chd4* were used as pool of two distinct shRNAs. Complete sequences of shRNAs and control are provided in Supplementary Table S1.

#### siRNAs

siMax siRNA 21 mers, obtained from Eurofin Genomics, were used for the gene silencing of *LUC* (5’-UACGACGAUUCUGUGAUUU-3’) as control and *CHD4* (siRNA sequences - si*CHD4*-1: 5’-CCCAG AAGAGGAUUUGUCA-3’ and si*CHD4*-2: 5’-GGUUU AAGCUCUUAGAACA-3’). siRNAs targeting *CHD4* were also used in pool.

### Cell cultures and infection

MCF10DCIS.com (obtained from Wayne State University, 5057 Woodward Avenue, Detroit - Michigan) and MCF10A (obtained from NIH Institute and authenticated in house by Gene Print 10 System, Promega) cell lines were maintained in their respective media as recommended by suppliers. MMTV-NeuT cells were obtained from dissociation of tumors following protocol described in www.stemcell.com. Human metastatic breast cancer (MBC) xenografts were obtained by direct implantation of patient-derived tumor tissue into the mammary fat pad of NOD.Cg-Prkdc^scid^ Il2rg^tm1Wjl^/SzJ mice (NSG). Patient–derived xenografted tumors (PDXs) were then serially re-transplanted to generate secondary tumors. PDX cells were obtained by enzymatic digestion and mechanical dissociation (Miltenyi Biotec) of tumors and grown in culture to obtain short-term human cultures. MMTV-NeuT and PDX cells were maintained in DMEM/F12 (1:1, Lonza/Gibco) supplemented with 10% Standard Fetal Bovine Serum (FBS) (HyClone, GE Healthcare Life Science), 10mM HEPES (Sigma), 5 μg/mL insulin (Roche), 0.5 μg/mL hydrocortisone (Sigma), 20 ng/mL (MMTV-NeuT cells) or 10 ng/mL (PDXs cells) epidermal growth factor (EGF, Tebu-Bio), 10 ng/mL (MMTV-NeuT cells) or 50 ng/mL (PDXs cells) Cholera Toxin (Sigma). Concentrated lentiviral particles (TU, transducing units) from libraries or single plasmids were either purchased by Cellecta Inc. or produced by transfecting 293T cells, as described in the Cellecta User Manual (http://www.cellecta.com/wp-content/uploads/Cellecta-Manual-13Kx13K-Barcode-Library-v1c.pdf). Lentiviral particles were added to MCF10DCIS.com, MCF10A, MMTV-NeuT or PDX short term cultures, together with 4 μg/mL polybrene (Sigma) for 16 hours. After 48 hours medium was replaced and 3μg/mL of puromycin was added for 72 hours before performing the experiments. Library infection was performed on MCF10DICS.com cells using a Multiplicity of Infection (MOI) of ~0.2 TU/cell. Conversely, in the *in vivo* validation and *in vitro* studies, cells were infected at high MOI (MCF10DCIS.com and MCF10A cells at MOI of ~3, MMTV-NeuT at MOI of ~20 and PDX culture cells at MOI of ~50 with pooled shRNAs silencing specific target genes).

### Animals

Non-obese diabetic/severe combined immunodeficiency (NOD/SCID) mice and Friend Virus B-Type (FVB) were purchased from Harlan Laboratories. NSG mice were purchased from Charles River. MMTV-NeuT transgenic mice were in the FVB background [71]. Only female mice 6-12 weeks old (15-20 gr weight) were used for experimental procedures.

### Ethics statement

Investigation has been conducted in accordance with the ethical standards and according to national and international guidelines. *In vivo* studies were performed after approval from our fully authorized animal facility, notification of the experiments to the Ministry of Health (as required by the Italian Law)(IACUCs N° 757/2015) and in accordance to EU directive 2010/63. Human tissue biopsies were collected from patients whose informed consent was obtained in writing according to the policies of the Ethics Committee of the European Institute of Oncology and regulations of Italian Ministry of Health. The studies were conducted in full compliance with the Declaration of Helsinki.

### *In vivo* and *in vitro* shRNA screens

1.2^10^6^ MCF10DCIS.com cells transduced with epigenetic libraries were orthotopically injected in the 4^th^ mammary gland of 12 NOD/SCID mice, or plated in duplicate *in vitro*. Reference cells, cells cultured for 21 days [72] and tumors harvested 28 days after transplantation [14] were subjected to DNA extraction. BCs representation was measured by Next Generation Sequencing (NGS) on Illumina HiSeq2000 and BCs were identified by aligning each sequencing read to the barcoded-libraries using the Bowtie aligner [73], and by considering only those BCs having, at most, three mismatches in each alignment. Detailed procedures of the analysis of the screen are described in Results. gDNA extraction, PCR assay and NGS were performed according to what reported in the Cellecta User Manual.

### *In vivo* study

MCF10DCIS.com, MMTV-NeuT and PDX cells were infected with control shRNA (sh*LUC*) and pooled shRNAs silencing specific target genes (see main text). 2.5^10^5^ infected MCF10DCIS.com or PDX cells and 5^10^5^ infected MMTV-NeuT cells were orthotopically injected into the 4^th^ mammary gland of 4 to 8 mice (respectively NOD/SCID, NSG or FVB). Tumor volume was calculated using this formula: V=l^2^^L/2 (l length; L width).

### *In vitro* studies

#### Proliferation assay

2^10^3^ MCF10DCIS.com or MCF10A infected cells were plated in triplicate (see above) and cell proliferation was measured by CellTiter-Glo assay (Promega). 8^10^4^ MMTV-NeuT infected cells were plated in triplicate and counted every 48 hours.

#### Migration assay

The migration assay was performed using 8.0μm pore size inserts in 24-well plates. Triplicates of 2.5^10^5^ MCF10DCIS.com cells were seeded in the upper chamber in 0.5% horse serum and complete medium supplemented with 50% FBS were added as chemoattractant in the lower chamber [74]. 5^10^4^ overnight starved (1% horse serum) MCF10A cells were seeded in the upper chamber and complete medium was added in the lower chamber. After 24 hours of incubation, migrated cells were fixed in 10% methanol and stained with 0.5% Crystal Violet. Migration was quantified by ImageJ analysis. Triplicates of 1^10^5^ MMTV-NeuT cells in growth factors and serum free medium were seeded in the upper chamber and complete medium supplemented with 50% FBS was used as chemoattractant. After 24 hours of incubation, cell migration was quantified as described previously.

#### Colony formation assay

Clonogenic potential of 1^10^3^ MCF10DCIS.com and MCF10A cells was measured in triplicates. After 7 days of culture, colonies were fixed, stained and counted as described above.

### Immunohistochemistry

Tumor fragments from PDXs were formalin-fixed and paraffin-embedded. After deparaffinization, sections were treated with 1 mM EDTA buffer (pH=8) for 30 min at 95°C, followed by incubation with 3% hydrogen peroxide in distilled water for 5 min at RT. Sections were stained with monoclonal anti-estrogen (ER) (Dako, clone 1D5); monoclonal anti-progesterone (PgR) (Dako, clone PgR 636); polyclonal anti-ErbB2 (Dako-A0485); monoclonal anti-Ki67 (Dako, clone MIB-1). Images were acquired by OLYMPUS BX51 up-right (objective UPIanAPO 20x/0,85) connected to Nikon Color Camera Digital Sight DS-U1 (software NIS-elements).

### Cell cycle analysis

#### BrdU content analysis

5 days post shRNA infection (sh*LUC* and sh*CHD4*), MCF10DCIS.com cells were pulsed with 5 mM Bromodeoxyuridine (BrdU), fixed and stained against BrdU (BD Biosciences). Pellet cells were stained with secondary antibody, incubated with propidium iodide (PI) and RNaseA and then acquired by fluorescent-activated cell sorting (FACS) at FACS Canto II (BD Bioscience). Analysis was performed using FlowJo 9.3-2 analysis software.

#### Multiparameter image cytometry

MCF10DCIS.com cells were transfected with siMax siRNA 21 mer silencing *LUC* or *CHD4* (as single siRNA: si*CHD4*-1 and si*CHD4*-2 and pooled siRNA: si*CHD4*-pool) using Lipofectamine RNAiMAX reagent (Life Technologies, 13778-075). After 72h, cells were pulsed with 10 μM EdU (a synthetic nucleotide to identify DNA-replicating cells), fixed and stained against CHD4 (Sigma-HPA012008), Ki67 (BD Pharmigen-558615), TP53 (Santa Cruz-sc6243), CDKN1A (Dako-M7202) and EdU (Click-iT™ Imaging kit; Life Technologies), according to manufacturer instructions. Images were collected by a BX61 fully motorized Olympus fluorescence microscope controlled by Scan^R software. An oil immersion 60X 1.3 NA objective was employed for acquisition. Cell cycle statistical analysis was performed as described by Furia and colleagues [61].

### Quantitative RT-PCR (qPCR)

Total RNA was extracted from MCF10DCIS.com cells infected with sh*CHD4* and sh*LUC* using the Quick-RNA MiniPrep kit ZymoResearch and reverse transcribed using EasyScript Plus Reverse Transcriptase and EasyScript Plus cDNA Synthesis kit. Quantitative RT-PCR analyses were done in triplicate on the Applied Biosystems 7500 Fast Real-Time PCR System with the fast-SYBR Green PCR kit as instructed by the manufacturer (Applied Biosystems). The transcription level of RPLP0 was used as housekeeper.

### Western blot analysis

MCF10DCIS.com, MCF10A, MMTV-NeuT and PDX cells were lysed in RIPA buffer supplemented with protease inhibitors (Roche). Protein extracts were resolved on SDS-polyacrylamide gel, blotted onto nitrocellulose membranes and probed with antibodies against Vinculin (Sigma V9131), BAZ1B (Abcam ab51256), BRD4 (Abcam ab128874), CHD4 (Abcam ab70469), BPTF (Novus Bio-NB100 41418). Membranes were incubated with appropriate secondary antibodies linked to horseradish peroxidase. Blots were then developed with the ECL system according to manufacturer's protocols and acquired by VueScan 9 x 32 (9.0.89). Images have been cropped at specific protein band of interest to improve the clarity of data presentation.

### Gene set enrichment analysis and protein interaction

The list of candidate hits scored in the *in vivo* and *in vitro* settings was uploaded to the Ingenuity Pathway Analysis (IPA) software (Qiagen, Valencia, CA) and IPA core analysis was run to analyze pathways and genes interaction scoring at high significance. Gene list was also investigated in Molecular Signature Data Base, MSigDB (http://software.broadinstitute.org/gsea/msigdb/annotate.jsp), applying “Compute Overlaps” tool and “Hallmark gene sets”. Protein interaction network was analyzed via online Search Tool for the Retrieval of Interacting Genes (STRING v 10.0 http://string-db.org).

### Statistical analysis

The correlations between tumors, cells and pools in the *in vivo* and *in vitro* screen were analyzed by Pearson correlation analysis. *In vitro* and *in vivo* data are presented as the mean ± s.d. (standard deviation) from three independent experiments. QPCR data are reported as mean ± s.e. (standard error) of two independent experiments done in triplicate. Statistical analyses were performed using a two-tailed Student's *t-*test and one way ANOVA plus post-hoc Dunnett's test. *In vivo* read out of MMTV-NeuT transplantation assay was analyzed with a *Mann*-*Whitney* U test. Differences were considered statistically significant at p<0.05 (*), p<0.01 (**) and p<0.001 (***).

## SUPPLEMENTARY MATERIALS FIGURES AND TABLES


