# Supplementary material for: RNAi screens identify CHD4 as an essential gene in breast cancer growth
Source: Oncotarget. 2016 Oct 13;7(49):80901–15. doi: 10.18632/oncotarget.12646 (PMC5348363; doi:10.18632/oncotarget.12646)
Supplement: Supplementary file 1 [file oncotarget-07-80901-s001.pdf]

RNAi screens identify CHD4 as an essential gene in breast cancer growth

SUPPLEMENTARY FIGURES AND TABLE

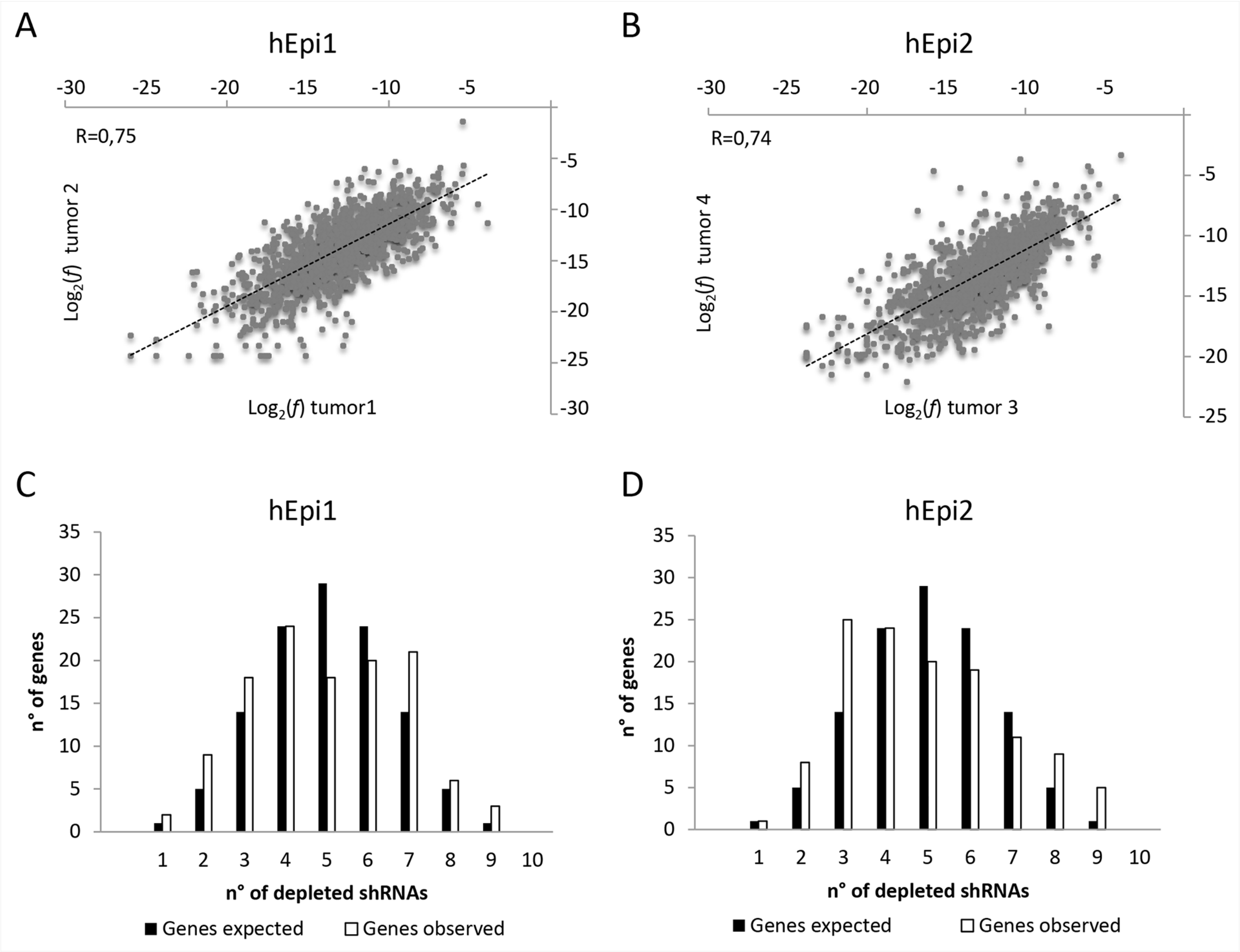

Supplementary Figure S1: Correlation between replicates analyzed as single or pool tumors. A, B. Scatter plot representation of shRNAs $\log_2(f)$ of two single tumors (tumor 1 *vs* 2 in A and 3 *vs* 4 in B) (out of four) arisen after transplantation of MCF10DCIS.com cell line infected with two distinct human epigenetic libraries, hEpi1 (A) and hEpi2 (B), respectively. Black dotted lines represent the axis bisectors. Pearson correlation coefficient (R) indicates the similarity between the samples analyzed. **C, D.** Histograms represent the comparison between the “observed genes” (white bars) and the “expected genes” (black bars) calculated by applying a hypergeometric distribution to the number of shRNAs (0 to 10) required to define a gene depleted. Each bar represents the probability to find a certain number of genes (y-axis) expected to be depleted (black bars) or the observed ones (white bars) in the screen at a given shRNA number (x-axis) in the two epigenetic libraries (hEpi1 – C and hEpi2 - D).

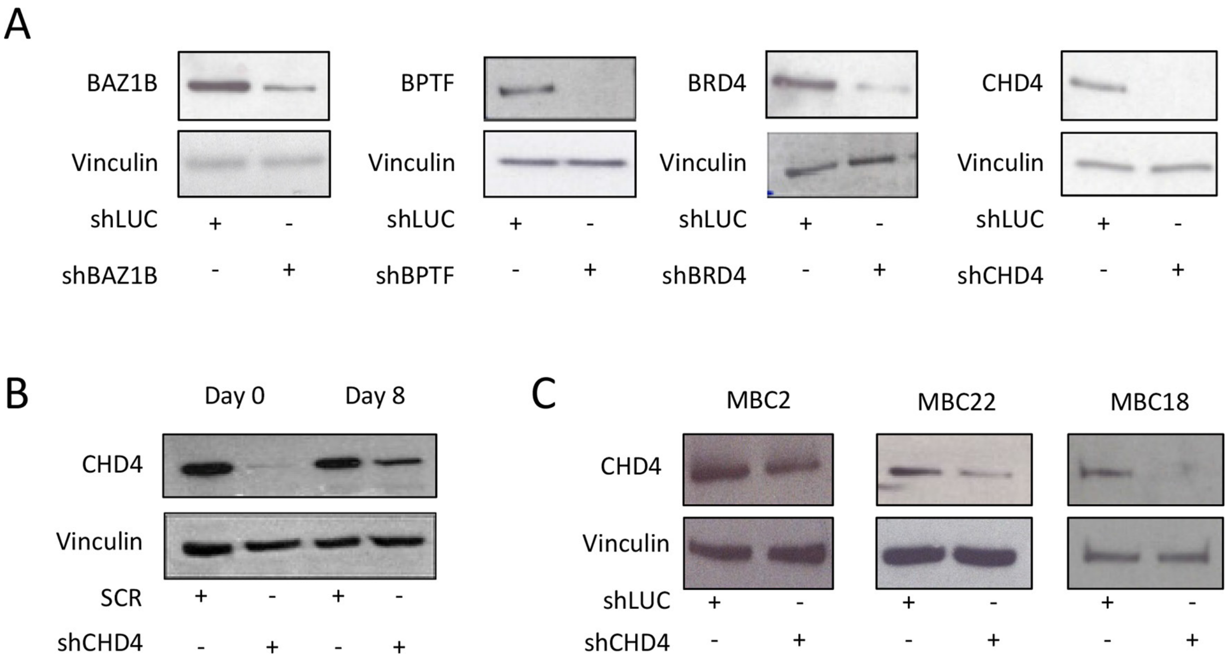

Supplementary Figure S2: Silencing efficiency of *BAZ1B*, *BPTF*, *BRD4* and *CHD4* shRNAs. A. MCF10DCIS.com cells infected with shRNAs targeting the candidate hits and the control (sh*LUC*) were subjected to western blot analysis. Cells were silenced using two pooled shRNAs/gene. **B.** Western blot analysis of CHD4 levels was performed on MMTV-NeuT cells silenced for *CHD4* or control (SCR) at two different time points. Lysates were collected immediately after puromycin selection (Day 0) and at the end of proliferation assay (Day 8). **C.** Cells derived from metastatic breast cancer (MBC) patient-derived xenografts were silenced for *CHD4* and protein level measured by western blot. Vinculin was used as normalizer.

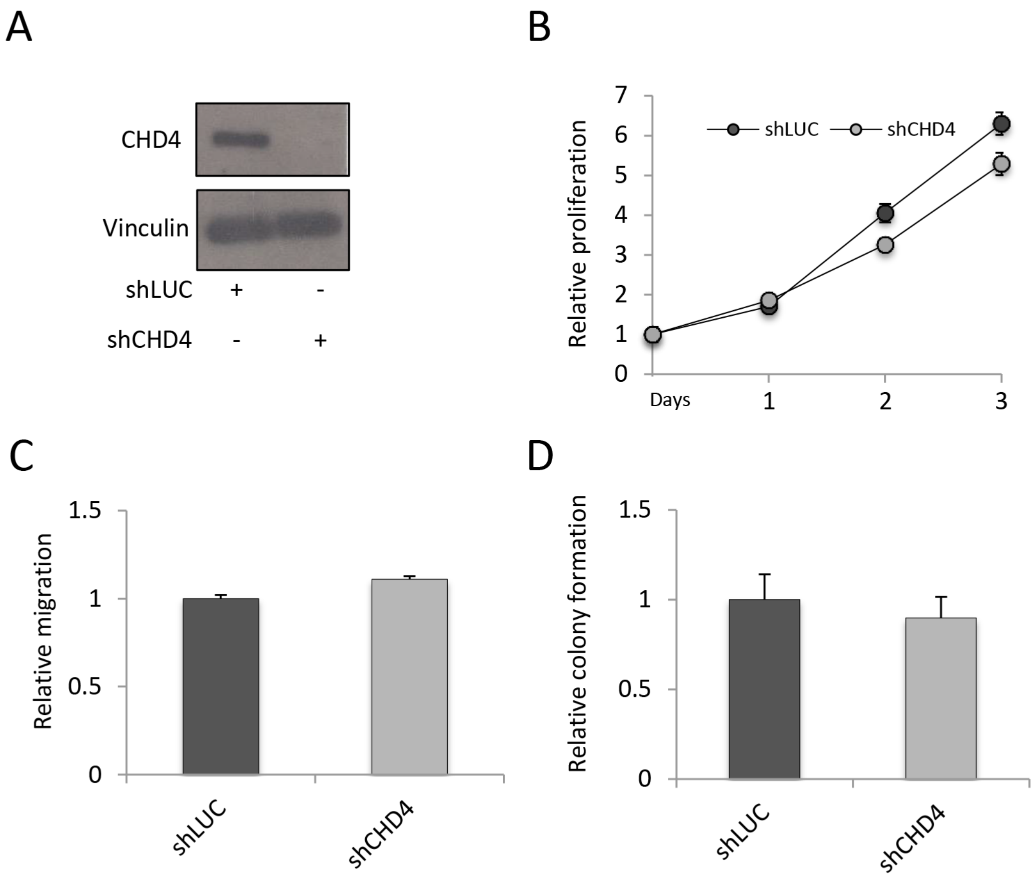

Supplementary Figure S3: Study of *CHD4* function in MCF10A cell line. MCF10A cell line was infected with a pool of shRNAs targeting *CHD4* or the neutral control (sh*LUC*). **A.** Western blot analysis of CHD4 level in MCF10A transduced cells. Vinculin was used as normalizer. Transduced mammary epithelial cells were used to analyze *in vitro* proliferative **B.** and migratory **C.** and clonogenic properties **D.**

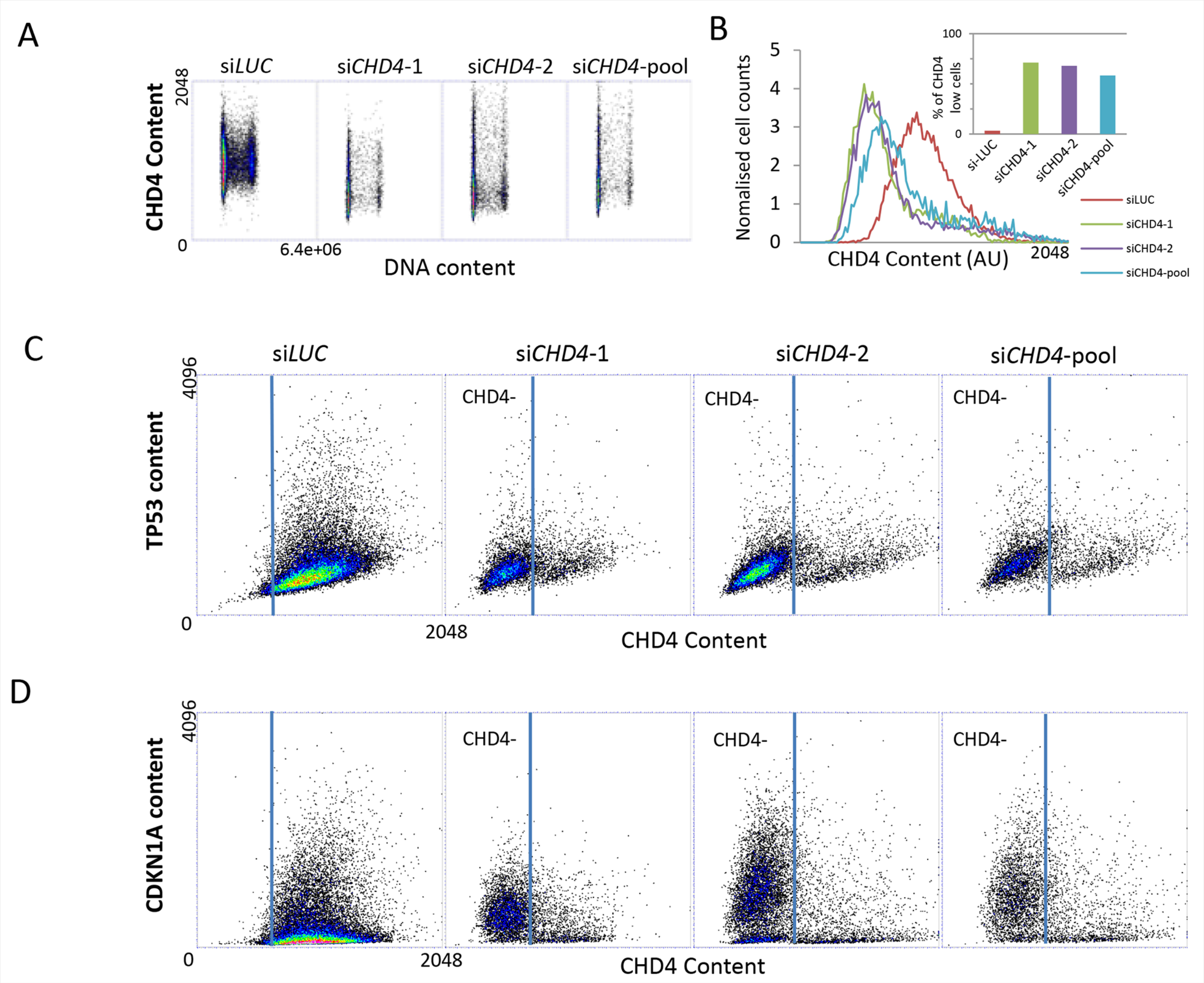

Supplementary Figure S4: Correlation of *CHD4* content and TP53 and CDKN1A levels in *CHD4* positive cells using high-content and high-resolution multiparameter image cytometry. MCF10DCIS.com cells transfected with two distinct (si*CHD4*-1 and si*CHD4*-2) or pooled (si*CHD4*-pool) siRNA against *CHD4* and the control (si*LUC*) were used to evaluate silencing efficacy, represented as *CHD4* content correlated to DNA content **A.** and as normalized cell count **B.** Curves represent the distribution of *CHD4* content in the cell populations (silenced and control cells). Inside histograms indicate the percentage of cells with low level of *CHD4* content. TP53 **C.** and CDKN1A **D.** levels were examined in MCF10DCIS.com transduced cells in correlation with *CHD4* content.

Supplementary Table S1: hEpi1 genes, functional class and depletion rank of genes according to shRNAs depletion and average of the z-score values between replicates of *in vivo* and *in vitro* screens. hEpi2 genes, functional class and depletion rank of genes according to shRNAs depletion and average of the z-score values between replicates of *in vivo* and *in vitro*. Sequences of shRNAs used for *in vivo* and *in vitro* screening validation of human epigenetic hits. Enrichment gene analysis performed on epigenetic hits scored in common between *in vivo* and *in vitro* screens.

See Supplementary File 1
